# [(1*R*,3*S*)-3-(1,3-Dithian-2-yl)-2,2-dimethyl­cyclo­prop­yl]diphenyl­methanol

**DOI:** 10.1107/S1600536809016079

**Published:** 2009-05-07

**Authors:** Risong Na, Min Wang

**Affiliations:** a Department of Applied Chemistry, China Agricultural University, 100193 Beijing, People’s Republic of China

## Abstract

In the title compound, C_22_H_26_OS_2_, prepared from (–)-1*R*-*cis*-caronaldehyde, the 1,3-dithiane ring adopts a chair conformation. An intra­molecular O—H⋯S hydrogen bond influences the mol­ecular conformation. In the crystal, weak inter­molecular C—H⋯S and C—H⋯O hydrogen bonds link the mol­ecules into chains propagating along [010].

## Related literature

For the details of preparation of the analogous compound, (1*R*,3*S*)-methyl-3-(1,3-dithian-2-yl)-2,2-dimethyl­cyclo­propane carboxyl­ate, see: Mazzanti *et al.* (1997 ▶); Veyrat *et al.* (1997 ▶); Perollier *et al.* (1997 ▶). 
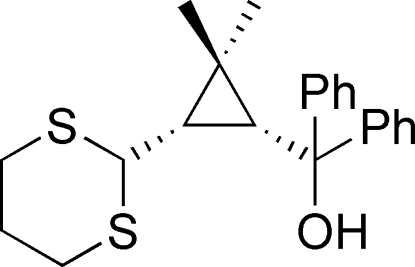


## Experimental

### 

#### Crystal data

C_22_H_26_OS_2_
*M* *_r_* = 370.55
Monoclinic, 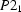

*a* = 9.5578 (19) Å
*b* = 11.199 (2) Å
*c* = 9.6512 (19) Å
β = 101.14 (3)°
*V* = 1013.6 (4) Å^3^
*Z* = 2
Mo *K*α radiation
μ = 0.27 mm^−1^
*T* = 123 K
0.40 × 0.40 × 0.30 mm

#### Data collection

Rigaku R-AXIS RAPID IP diffractometer
Absorption correction: multi-scan (*ABSCOR*; Higashi, 1995 ▶) *T*_min_ = 0.900, *T*_max_ = 0.924
4303 measured reflections
4303 independent reflections
2920 reflections with *I* > 2σ(*I*)

#### Refinement

*R*[*F* ^2^ > 2σ(*F* ^2^)] = 0.038
*wR*(*F* ^2^) = 0.084
*S* = 0.84
4303 reflections
229 parameters
1 restraint
H-atom parameters constrained
Δρ_max_ = 0.28 e Å^−3^
Δρ_min_ = −0.30 e Å^−3^
Absolute structure: Flack (1983 ▶), 1878 Friedel pairs
Flack parameter: 0.05 (7)

### 

Data collection: *RAPID-AUTO* (Rigaku, 2000 ▶); cell refinement: *RAPID-AUTO*; data reduction: *CrystalStructure* (Rigaku, 2000 ▶); program(s) used to solve structure: *SHELXS97* (Sheldrick, 2008 ▶); program(s) used to refine structure: *SHELXL97* (Sheldrick, 2008 ▶); molecular graphics: *SHELXTL* (Sheldrick, 2008 ▶); software used to prepare material for publication: *SHELXL97*.

## Supplementary Material

Crystal structure: contains datablocks I, global. DOI: 10.1107/S1600536809016079/cv2542sup1.cif

Structure factors: contains datablocks I. DOI: 10.1107/S1600536809016079/cv2542Isup2.hkl

Additional supplementary materials:  crystallographic information; 3D view; checkCIF report

## Figures and Tables

**Table 1:** Hydrogen-bond geometry (Å, °)

*D*—H⋯*A*	*D*—H	H⋯*A*	*D*⋯*A*	*D*—H⋯*A*
O1—H2⋯S1	0.84	2.58	3.330 (2)	149
C7—H7*A*⋯S2^i^	0.99	2.89	3.736 (3)	144
C9—H9*A*⋯O1^ii^	0.99	2.60	3.236 (3)	122

Symmetry codes: (i) 
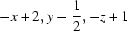
; (ii) 
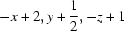
.

**Fig. 1.**
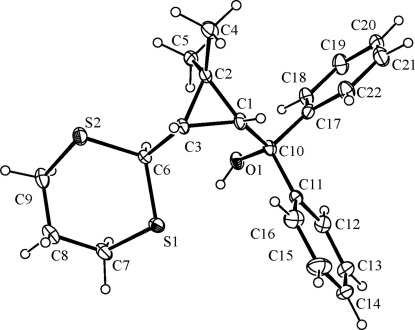
The molecular structure of (I) showing the atomic numbering and 30% probability displacement ellipsoids for non-H atoms.

## supplementary crystallographic information

### Comment

We designed and prepared a novel type of chiral dithiane alcohol based with chiral *cis*-cyclopropane from (-)-1*R*-*cis*-Caronaldehyde. Details of preparation of the analogue compound were descussed in the literature (Mazzanti *et al.*, 1997; Veyrat *et al.*, 1997; Perollier *et al.*, 1997).

In this paper, the crystal structure of the title compound, (I), is reported. In (I) (Fig. 1), the 1,3-dithiane ring adopts a chair conformation. Intramolecular O—H···S hydrogen bond (Table 1) influences the molecular conformation. In the crystal,  weak intermolecular C—H···O and C—H···S hydrogen bonds (Table 1) link the molecules into chains propagated in direction [010].

### Experimental

Magnesium (0.4 g, 15.6 mmol) was added to 15 ml of anhydrous THF. A solution of bromobenzene (2.0 g, 12.5 mmol in 5 ml of THF) was added dropwise into the above mixture. Once the reaction began, the rest of the bromobenzene solution was added at a rate that maintained a gentle reflux. When the addition of the bromobenzene solution was complete, the mixture was refluxed for 20 min, and was then cooled to 273 K. (1*R*,3*S*)-Methyl-3-(1,3-dithian-2-yl)-2,2-dimethylcyclopropane carboxylate (5 mmol) (Mazzanti *et al.*, 1997; Veyrat *et al.*, 1997; Perollier *et al.*, 1997) was dissolved in 5 ml of anhydrous THF and added to the prepared Grignard mixture. After the solution of carboxylate had been added, the resulting mixture was stirred at room temperature for an additional 24 h. The reaction was quenched with saturated NH_4_Cl (aq), and the mixture was extracted several times with Et_2_O. The organic phases were combined, dried over MgSO_4_ and concentrated under reduced pressure. The residual yellow solid was purified by recrystallization in Et_2_O to yield compound (I) as colourless crystals. Colourless solid, m.p. 427 K; [α]_20_^D^ =+19.93 (*c* 0.03, CHCl_3_).

### Refinement

All H atoms were positioned geometrically and treated as riding on their parent atoms with C—H = 0.95 (C_aromatic_), 0.98 (C_methyl_), 0.99 (CH_2_) and 1.00 (CH) Å  and O—H = 0.84 Å, and with *U*_iso_ (H) = 1.5*U*_eq_ (C_methy_,*O*) and 1.2*U*_eq_ (other C).

### Crystal data

**Table:**

C_22_H_26_OS_2_	*F*(000) = 396
*M**_r_* = 370.55	*D*_x_ = 1.214 Mg m^−^^3^
Monoclinic, *P*2_1_	Mo *K*α radiation, λ = 0.71073 Å
Hall symbol: P 2yb	Cell parameters from 7560 reflections
*a* = 9.5578 (19) Å	θ = 2.2–27.5°
*b* = 11.199 (2) Å	µ = 0.27 mm^−^^1^
*c* = 9.6512 (19) Å	*T* = 123 K
β = 101.14 (3)°	Block, colourless
*V* = 1013.6 (4) Å^3^	0.40 × 0.40 × 0.30 mm
*Z* = 2	

### Data collection

**Table:**

Rigaku R-AXIS RAPID IP diffractometer	4303 independent reflections
Radiation source: fine-focus sealed tube	2920 reflections with *I* > 2σ(*I*)
graphite	*R*_int_ = 0.0000
Detector resolution: 10.00 pixels mm^-1^	θ_max_ = 27.5°, θ_min_ = 2.2°
ω scans	*h* = −12→12
Absorption correction: multi-scan (*ABSCOR*; Higashi, 1995)	*k* = −14→14
*T*_min_ = 0.900, *T*_max_ = 0.924	*l* = −12→12
4303 measured reflections	

### Refinement

**Table:**

Refinement on *F*^2^	Hydrogen site location: inferred from neighbouring sites
Least-squares matrix: full	H-atom parameters constrained
*R*[*F*^2^ > 2σ(*F*^2^)] = 0.038	*w* = 1/[σ^2^(*F*_o_^2^) + (0.0379*P*)^2^] where *P* = (*F*_o_^2^ + 2*F*_c_^2^)/3
*wR*(*F*^2^) = 0.084	(Δ/σ)_max_ = 0.001
*S* = 0.84	Δρ_max_ = 0.28 e Å^−^^3^
4303 reflections	Δρ_min_ = −0.30 e Å^−^^3^
229 parameters	Extinction correction: *SHELXL97* (Sheldrick, 2008), Fc^*^=kFc[1+0.001xFc^2^λ^3^/sin(2θ)]^-1/4^
1 restraint	Extinction coefficient: 0.046 (2)
Primary atom site location: structure-invariant direct methods	Absolute structure: Flack (1983), 1878 Friedel pairs
Secondary atom site location: difference Fourier map	Flack parameter: 0.05 (7)

### Special details

**Table:**

Geometry. All e.s.d.'s (except the e.s.d. in the dihedral angle between two l.s. planes) are estimated using the full covariance matrix. The cell e.s.d.'s are taken into account individually in the estimation of e.s.d.'s in distances, angles and torsion angles; correlations between e.s.d.'s in cell parameters are only used when they are defined by crystal symmetry. An approximate (isotropic) treatment of cell e.s.d.'s is used for estimating e.s.d.'s involving l.s. planes.
Refinement. Refinement of *F*^2^ against ALL reflections. The weighted *R*-factor *wR* and goodness of fit *S* are based on *F*^2^, conventional *R*-factors *R* are based on *F*, with *F* set to zero for negative *F*^2^. The threshold expression of *F*^2^ > σ(*F*^2^) is used only for calculating *R*-factors(gt) *etc*. and is not relevant to the choice of reflections for refinement. *R*-factors based on *F*^2^ are statistically about twice as large as those based on *F*, and *R*- factors based on ALL data will be even larger.

### Fractional atomic coordinates and isotropic or equivalent isotropic displacement parameters (Å^2^)

**Table:**

	*x*	*y*	*z*	*U*_iso_*/*U*_eq_	
C1	0.6857 (3)	0.2287 (2)	0.2178 (3)	0.0254 (6)	
H1	0.6517	0.2825	0.1355	0.030*	
C2	0.6466 (3)	0.2775 (2)	0.3516 (3)	0.0287 (7)	
C3	0.8003 (3)	0.2893 (2)	0.3280 (3)	0.0238 (6)	
H3	0.8246	0.3717	0.3005	0.029*	
C4	0.5595 (3)	0.3923 (3)	0.3379 (3)	0.0462 (9)	
H4A	0.4577	0.3727	0.3176	0.069*	
H4B	0.5827	0.4407	0.2609	0.069*	
H4C	0.5822	0.4373	0.4265	0.069*	
C5	0.6172 (3)	0.1950 (3)	0.4668 (3)	0.0369 (8)	
H5A	0.5156	0.1745	0.4492	0.055*	
H5B	0.6432	0.2350	0.5585	0.055*	
H5C	0.6739	0.1220	0.4674	0.055*	
C6	0.9233 (2)	0.2264 (2)	0.4217 (3)	0.0236 (6)	
H6	0.8867	0.1549	0.4656	0.028*	
C7	1.1962 (3)	0.1241 (3)	0.4492 (3)	0.0351 (7)	
H7A	1.1632	0.0529	0.4944	0.042*	
H7B	1.2751	0.0987	0.4028	0.042*	
C8	1.2536 (3)	0.2153 (2)	0.5636 (3)	0.0354 (8)	
H8A	1.3404	0.1827	0.6245	0.042*	
H8B	1.2811	0.2887	0.5184	0.042*	
C9	1.1470 (3)	0.2472 (3)	0.6542 (3)	0.0354 (7)	
H9A	1.1958	0.2940	0.7366	0.042*	
H9B	1.1109	0.1729	0.6902	0.042*	
C10	0.6776 (3)	0.0972 (2)	0.1726 (3)	0.0235 (6)	
C11	0.7437 (3)	0.0754 (2)	0.0414 (3)	0.0258 (6)	
C12	0.7434 (3)	−0.0429 (3)	−0.0102 (3)	0.0389 (8)	
H12	0.6995	−0.1044	0.0341	0.047*	
C13	0.8053 (3)	−0.0705 (3)	−0.1232 (3)	0.0479 (9)	
H13	0.8059	−0.1507	−0.1551	0.057*	
C14	0.8676 (3)	0.0196 (4)	−0.1912 (3)	0.0522 (10)	
H14	0.9091	0.0014	−0.2705	0.063*	
C15	0.8679 (4)	0.1346 (3)	−0.1422 (4)	0.0537 (10)	
H15	0.9100	0.1958	−0.1885	0.064*	
C16	0.8079 (3)	0.1635 (3)	−0.0258 (3)	0.0404 (8)	
H16	0.8108	0.2435	0.0074	0.048*	
C17	0.5212 (3)	0.0589 (2)	0.1427 (3)	0.0259 (6)	
C18	0.4713 (3)	−0.0319 (3)	0.2171 (3)	0.0345 (7)	
H18	0.5356	−0.0737	0.2880	0.041*	
C19	0.3262 (3)	−0.0628 (3)	0.1886 (3)	0.0412 (8)	
H19	0.2933	−0.1268	0.2385	0.049*	
C20	0.2309 (3)	−0.0007 (3)	0.0882 (3)	0.0392 (8)	
H20	0.1323	−0.0203	0.0713	0.047*	
C21	0.2790 (3)	0.0895 (3)	0.0129 (3)	0.0410 (8)	
H21	0.2141	0.1313	−0.0575	0.049*	
C22	0.4234 (3)	0.1196 (3)	0.0402 (3)	0.0370 (8)	
H22	0.4559	0.1825	−0.0117	0.044*	
O1	0.75071 (18)	0.02068 (15)	0.28301 (18)	0.0294 (5)	
H2	0.8381	0.0324	0.2863	0.035*	
S1	1.05026 (7)	0.17887 (6)	0.31479 (7)	0.02959 (18)	
S2	0.99674 (7)	0.33324 (6)	0.55984 (8)	0.0331 (2)	

### Atomic displacement parameters (Å^2^)

**Table:**

	*U*^11^	*U*^22^	*U*^33^	*U*^12^	*U*^13^	*U*^23^
C1	0.0253 (14)	0.0246 (14)	0.0234 (16)	0.0024 (12)	−0.0025 (12)	0.0004 (11)
C2	0.0194 (14)	0.0317 (15)	0.0309 (17)	0.0023 (12)	−0.0056 (12)	−0.0089 (13)
C3	0.0195 (13)	0.0240 (14)	0.0248 (15)	0.0010 (11)	−0.0030 (11)	−0.0005 (11)
C4	0.0302 (17)	0.0495 (19)	0.053 (2)	0.0138 (15)	−0.0065 (15)	−0.0172 (16)
C5	0.0221 (14)	0.058 (2)	0.0314 (16)	−0.0069 (14)	0.0060 (12)	−0.0125 (16)
C6	0.0179 (13)	0.0270 (14)	0.0247 (15)	0.0028 (11)	0.0008 (11)	−0.0022 (11)
C7	0.0208 (15)	0.0375 (17)	0.045 (2)	−0.0002 (12)	0.0011 (13)	−0.0007 (14)
C8	0.0220 (15)	0.0344 (18)	0.046 (2)	−0.0034 (13)	−0.0026 (14)	0.0076 (14)
C9	0.0328 (16)	0.0335 (16)	0.0338 (18)	−0.0009 (14)	−0.0089 (14)	−0.0005 (13)
C10	0.0230 (14)	0.0218 (14)	0.0230 (15)	0.0046 (12)	−0.0021 (11)	0.0027 (11)
C11	0.0221 (14)	0.0318 (15)	0.0216 (14)	0.0074 (13)	−0.0008 (11)	0.0014 (12)
C12	0.0363 (18)	0.0429 (18)	0.0362 (18)	0.0034 (15)	0.0036 (14)	−0.0071 (15)
C13	0.0389 (19)	0.068 (3)	0.036 (2)	0.0096 (18)	0.0057 (16)	−0.0185 (19)
C14	0.0364 (19)	0.093 (3)	0.0271 (18)	0.021 (2)	0.0066 (15)	−0.013 (2)
C15	0.052 (2)	0.069 (3)	0.047 (2)	0.0117 (18)	0.0268 (18)	0.0166 (18)
C16	0.0431 (18)	0.0439 (19)	0.0360 (18)	0.0079 (17)	0.0121 (14)	0.0093 (16)
C17	0.0306 (15)	0.0253 (15)	0.0207 (14)	0.0019 (12)	0.0027 (12)	−0.0046 (12)
C18	0.0322 (16)	0.0349 (17)	0.0321 (17)	−0.0011 (14)	−0.0044 (13)	0.0055 (13)
C19	0.0405 (19)	0.0399 (18)	0.0406 (19)	−0.0124 (15)	0.0013 (15)	0.0031 (15)
C20	0.0277 (16)	0.050 (2)	0.0367 (19)	−0.0058 (15)	−0.0003 (14)	−0.0071 (15)
C21	0.0308 (17)	0.051 (2)	0.0378 (19)	0.0016 (16)	−0.0032 (14)	0.0019 (15)
C22	0.0334 (17)	0.0415 (18)	0.0338 (18)	−0.0031 (14)	0.0005 (14)	0.0093 (14)
O1	0.0285 (11)	0.0267 (10)	0.0287 (11)	0.0028 (8)	−0.0053 (9)	0.0036 (9)
S1	0.0226 (3)	0.0370 (4)	0.0294 (4)	0.0013 (3)	0.0058 (3)	0.0009 (3)
S2	0.0289 (4)	0.0306 (4)	0.0356 (4)	0.0023 (3)	−0.0043 (3)	−0.0068 (4)

### Geometric parameters (Å, °)

**Table:**

C1—C2	1.515 (4)	C9—H9B	0.9900
C1—C3	1.531 (3)	C10—O1	1.439 (3)
C1—C10	1.533 (4)	C10—C17	1.529 (4)
C1—H1	1.0000	C10—C11	1.540 (4)
C2—C5	1.513 (4)	C11—C16	1.387 (4)
C2—C4	1.523 (4)	C11—C12	1.415 (4)
C2—C3	1.536 (4)	C12—C13	1.372 (4)
C3—C6	1.511 (3)	C12—H12	0.9500
C3—H3	1.0000	C13—C14	1.398 (5)
C4—H4A	0.9800	C13—H13	0.9500
C4—H4B	0.9800	C14—C15	1.371 (5)
C4—H4C	0.9800	C14—H14	0.9500
C5—H5A	0.9800	C15—C16	1.395 (4)
C5—H5B	0.9800	C15—H15	0.9500
C5—H5C	0.9800	C16—H16	0.9500
C6—S1	1.818 (3)	C17—C18	1.382 (4)
C6—S2	1.827 (3)	C17—C22	1.399 (4)
C6—H6	1.0000	C18—C19	1.404 (4)
C7—C8	1.526 (4)	C18—H18	0.9500
C7—S1	1.818 (3)	C19—C20	1.382 (4)
C7—H7A	0.9900	C19—H19	0.9500
C7—H7B	0.9900	C20—C21	1.375 (4)
C8—C9	1.508 (4)	C20—H20	0.9500
C8—H8A	0.9900	C21—C22	1.396 (4)
C8—H8B	0.9900	C21—H21	0.9500
C9—S2	1.821 (3)	C22—H22	0.9500
C9—H9A	0.9900	O1—H2	0.8400
			
C2—C1—C3	60.55 (17)	S2—C9—H9A	109.0
C2—C1—C10	125.6 (2)	C8—C9—H9B	109.0
C3—C1—C10	127.8 (2)	S2—C9—H9B	109.0
C2—C1—H1	111.3	H9A—C9—H9B	107.8
C3—C1—H1	111.3	O1—C10—C17	106.8 (2)
C10—C1—H1	111.3	O1—C10—C1	111.8 (2)
C5—C2—C1	121.2 (2)	C17—C10—C1	108.5 (2)
C5—C2—C4	113.8 (3)	O1—C10—C11	107.24 (19)
C1—C2—C4	116.8 (2)	C17—C10—C11	110.0 (2)
C5—C2—C3	118.7 (2)	C1—C10—C11	112.4 (2)
C1—C2—C3	60.24 (18)	C16—C11—C12	118.2 (3)
C4—C2—C3	115.9 (2)	C16—C11—C10	124.1 (2)
C6—C3—C1	125.3 (2)	C12—C11—C10	117.7 (2)
C6—C3—C2	121.8 (2)	C13—C12—C11	121.2 (3)
C1—C3—C2	59.21 (17)	C13—C12—H12	119.4
C6—C3—H3	113.3	C11—C12—H12	119.4
C1—C3—H3	113.3	C12—C13—C14	119.9 (3)
C2—C3—H3	113.3	C12—C13—H13	120.0
C2—C4—H4A	109.5	C14—C13—H13	120.0
C2—C4—H4B	109.5	C15—C14—C13	119.2 (3)
H4A—C4—H4B	109.5	C15—C14—H14	120.4
C2—C4—H4C	109.5	C13—C14—H14	120.4
H4A—C4—H4C	109.5	C14—C15—C16	121.5 (3)
H4B—C4—H4C	109.5	C14—C15—H15	119.3
C2—C5—H5A	109.5	C16—C15—H15	119.3
C2—C5—H5B	109.5	C11—C16—C15	120.0 (3)
H5A—C5—H5B	109.5	C11—C16—H16	120.0
C2—C5—H5C	109.5	C15—C16—H16	120.0
H5A—C5—H5C	109.5	C18—C17—C22	118.3 (3)
H5B—C5—H5C	109.5	C18—C17—C10	122.1 (2)
C3—C6—S1	108.90 (19)	C22—C17—C10	119.5 (2)
C3—C6—S2	106.04 (18)	C17—C18—C19	120.4 (3)
S1—C6—S2	113.65 (13)	C17—C18—H18	119.8
C3—C6—H6	109.4	C19—C18—H18	119.8
S1—C6—H6	109.4	C20—C19—C18	120.4 (3)
S2—C6—H6	109.4	C20—C19—H19	119.8
C8—C7—S1	114.18 (19)	C18—C19—H19	119.8
C8—C7—H7A	108.7	C21—C20—C19	119.9 (3)
S1—C7—H7A	108.7	C21—C20—H20	120.1
C8—C7—H7B	108.7	C19—C20—H20	120.1
S1—C7—H7B	108.7	C20—C21—C22	119.8 (3)
H7A—C7—H7B	107.6	C20—C21—H21	120.1
C9—C8—C7	112.9 (2)	C22—C21—H21	120.1
C9—C8—H8A	109.0	C21—C22—C17	121.2 (3)
C7—C8—H8A	109.0	C21—C22—H22	119.4
C9—C8—H8B	109.0	C17—C22—H22	119.4
C7—C8—H8B	109.0	C10—O1—H2	105.8
H8A—C8—H8B	107.8	C7—S1—C6	101.43 (13)
C8—C9—S2	113.0 (2)	C9—S2—C6	99.98 (13)
C8—C9—H9A	109.0		
			
C3—C1—C2—C5	−107.5 (3)	C1—C10—C11—C12	179.7 (2)
C10—C1—C2—C5	10.0 (4)	C16—C11—C12—C13	0.4 (4)
C3—C1—C2—C4	106.0 (3)	C10—C11—C12—C13	−177.4 (3)
C10—C1—C2—C4	−136.5 (3)	C11—C12—C13—C14	−1.4 (4)
C10—C1—C2—C3	117.5 (3)	C12—C13—C14—C15	1.1 (5)
C2—C1—C3—C6	109.2 (3)	C13—C14—C15—C16	0.1 (5)
C10—C1—C3—C6	−4.9 (4)	C12—C11—C16—C15	0.9 (4)
C10—C1—C3—C2	−114.1 (3)	C10—C11—C16—C15	178.5 (3)
C5—C2—C3—C6	−3.5 (4)	C14—C15—C16—C11	−1.1 (5)
C1—C2—C3—C6	−115.0 (3)	O1—C10—C17—C18	1.3 (3)
C4—C2—C3—C6	137.5 (3)	C1—C10—C17—C18	−119.4 (3)
C5—C2—C3—C1	111.5 (3)	C11—C10—C17—C18	117.4 (3)
C4—C2—C3—C1	−107.5 (3)	O1—C10—C17—C22	179.1 (2)
C1—C3—C6—S1	71.5 (3)	C1—C10—C17—C22	58.5 (3)
C2—C3—C6—S1	144.1 (2)	C11—C10—C17—C22	−64.8 (3)
C1—C3—C6—S2	−165.9 (2)	C22—C17—C18—C19	0.8 (4)
C2—C3—C6—S2	−93.3 (3)	C10—C17—C18—C19	178.6 (3)
S1—C7—C8—C9	66.4 (3)	C17—C18—C19—C20	−1.7 (5)
C7—C8—C9—S2	−69.4 (3)	C18—C19—C20—C21	1.9 (5)
C2—C1—C10—O1	−51.7 (3)	C19—C20—C21—C22	−1.3 (5)
C3—C1—C10—O1	26.2 (4)	C20—C21—C22—C17	0.4 (5)
C2—C1—C10—C17	65.9 (3)	C18—C17—C22—C21	−0.2 (4)
C3—C1—C10—C17	143.8 (3)	C10—C17—C22—C21	−178.1 (3)
C2—C1—C10—C11	−172.3 (2)	C8—C7—S1—C6	−55.9 (2)
C3—C1—C10—C11	−94.4 (3)	C3—C6—S1—C7	173.11 (18)
O1—C10—C11—C16	−121.1 (3)	S2—C6—S1—C7	55.16 (17)
C17—C10—C11—C16	123.0 (3)	C8—C9—S2—C6	61.1 (2)
C1—C10—C11—C16	2.1 (3)	C3—C6—S2—C9	−176.75 (19)
O1—C10—C11—C12	56.5 (3)	S1—C6—S2—C9	−57.17 (18)
C17—C10—C11—C12	−59.4 (3)		

### Hydrogen-bond geometry (Å, °)

**Table:**

*D*—H···*A*	*D*—H	H···*A*	*D*···*A*	*D*—H···*A*
O1—H2···S1	0.84	2.58	3.330 (2)	149
C7—H7A···S2^i^	0.99	2.89	3.736 (3)	144
C9—H9A···O1^ii^	0.99	2.60	3.236 (3)	122

Symmetry codes: (i) −*x*+2, *y*−1/2, −*z*+1; (ii) −*x*+2, *y*+1/2, −*z*+1.
